# Serologic evidence of orthomarburgviruses and an orthoebolavirus in frugivorous Malagasy bats

**DOI:** 10.1186/s42522-025-00178-0

**Published:** 2025-10-30

**Authors:** Marana S. Rekedal, Emily Cornelius Ruhs, Hafaliana Christian Ranaivoson, Neil Mittal, Spencer L. Sterling, Gwenddolen Kettenburg, Angelo Andrianiaina, Santino Andry, Lianying Yan, Axel T. Lehrer, Jean-Michel Héraud, Vincent Lacoste, Philippe Dussart, Cara E. Brook, Eric D. Laing

**Affiliations:** 1 Uniformed Services University of Health Sciences, Bethesda, MD USA; 2 The Henry M. Jackson Foundation for the Advancement of Military Medicine, Inc, Rockledge, MD USA; 3 University of Chicago, Chicago, IL USA; 4 The University of Antananarivo, Antananarivo, Madagascar; 5 University of Hawaiʻi at Mānoa, Honolulu, HI USA; 6 Institut Pasteur de Madagascar, Antananarivo, Madagascar; 7 Present Address: University of California, Berkeley, CA US

**Keywords:** Marburg virus, Bats, Surveillance, Madagascar, Filoviruses

## Abstract

Recent outbreaks of Marburg virus disease in regions of sub-Saharan Africa without prior record such as Ghana, Tanzania, Equatorial Guinea, and Rwanda challenge the current understanding of areas at-risk for filovirus spillover. The orthomarburgvirus host *Rousettus aegyptiacus* exists in these regions, and has a sister species, *Rousettus madagascariensis*, native only to the island of Madagascar. We expand on previous surveillance of frugivorous bats in Madagascar, and detect seroreactivity against an orthoebolavirus antigenically-related to Bundibugyo virus at 1.50–5.06% seroprevalence in *Rousettus madagascariensis*. Notably, we identify *R. madagascariensis* as an orthomarburgvirus-host species of interest in Madagascar, the first record of this virus genus on the island. We document seroprevalences of 15.36–29.78% for Ravn virus and 12.55–33.33% for Marburg virus in *R. madagascariensis*. These findings will guide future biosurveillance efforts to assess filovirus spillover risk in Madagascar.

**Supplementary Information:**

The online version contains supplementary material available at 10.1186/s42522-025-00178-0.

## Introduction

Pre-pandemic preparedness against filoviruses require accurate biosurveillance to identify region-specific risks for virus emergence. Marburg virus (MARV), one virus within the family *Filoviridae*, causes fatal outbreaks of viral hemorrhagic disease known as Marburg virus disease (MVD) in humans. Forty-two years after the first recorded MARV spillover event, molecular isolation of MARV and its relative Ravn virus (RAVV) and subsequent experimental challenge studies confirmed the Egyptian rousette bat (*Rousettus aegyptiacus)* as a natural host for orthomarburgviruses [1–6]. MVD outbreaks first occurred in South Africa (exported from Zimbabwe), Kenya, Angola, Uganda, and the Democratic Republic of the Congo, and have been historically associated with activities such as cave tourism and mining that placed humans in close proximity to cave-roosting *R. aegyptiacus* bats [7]. Since 2021, MVD outbreaks in Guinea, Ghana, Equatorial Guinea, and Tanzania have highlighted new regions at-risk for MVD [8–11]. On September 27th, 2024, the Republic of Rwanda confirmed an active MVD outbreak, initially misdiagnosed as malaria due to the country’s lack of prior MVD history. The outbreak ended in December with a total of 66 cases and 15 deaths [12]. In January of 2025, the Kagera region of the United Republic of Tanzania reported another outbreak of MVD, with ten fatal suspected cases by February 10th, further demonstrating the increasing frequency of recorded MARV infections [13]. *R. aegyptiacus* are endemic to both Rwanda and Tanzania, and have a broad geographic range, spanning Guinea to Pakistan and ranging in latitude from South Africa to Turkey.

In contrast to orthomarburgviruses, identification of the natural hosts of viruses of the *Filoviridae* family, genus *Orthoebolavirus*, has proven difficult. Although bats have been suggested as the natural hosts of orthoebolaviruses, the primary animal hosts and zoonotic transmission chains for Ebola virus (EBOV), Bundibugyo virus (BDBV), Sudan virus (SUDV), and Taï Forest virus (TAFV) remain unknown [14]. Definitive identification of natural reservoirs for orthoebolaviruses requires further detection of nucleic acid material, virus isolation, and experimental infection models; however, due to the limitations of this approach, serological detection of antibodies is frequently used in conjunction to detect history of virus infection in potential bat hosts [15–19].

Virus-host taxa predictions suggest that frugivorous bats in the genus *Rousettus* have a high probability for being wildlife hosts of viruses in the family *Filoviridae* [20, 21]. The *Rousettus* genus contains seven described species, with four species native to Asia and three to Africa. Of the Asiatic species, *R. leschanaulti* and *R. amplexicaudatus* are found across South and Southeast Asia, and are the likely hosts of newly identified filoviruses Měnglà virus (MLAV) and Dehong virus (DEHV), both of which constitute their own distinct viral genera [22, 23]. Serological evidence of an orthoebolavirus, Reston virus (RESTV), was detected in *R. amplexicaudatus* bats sampled in the Philippines [24], while molecular evidence of RESTV has also been found in *Miniopterus schreibersii* insectivorous bats and *Acerodon jubatus* flying foxes [25]. Of the African *Rousettus* species, two remain geographically restricted, with the Comoros rousette (*R. obliviosus*) found only in the Comoros Islands and the Madagascan rousette bat (*R. madagascariensis*) native to the island of Madagascar.

Orthoebolavirus seropositivity has been detected in species of *Pteropus* and *Eidolon* bats sampled in the Philippines, Cameroon, and the Democratic Republic of the Congo [17, 18, 25]. In Madagascar, previous virus surveillance of three native fruit bat species, *R. madagascariensis*,* Pteropus rufus*, and *Eidolon dupreanum*, detected serologic evidence of a virus antigenically-similar to EBOV in both *R. madagascariensis* and *P. rufus* bats [26]. In this study, we examined new samples collected from these three species with an expanded filovirus panel including eight different virus targets to investigate serological evidence of orthoebolaviruses and orthomarburgviruses circulating in this system and identify likely wildlife host species. We hypothesized that seroreactivity against orthomarburgviruses would be detected in *R. madagascariensis* given the historical association of the *Rousettus* genus with orthomarburgviruses. To test that hypothesis, we comparatively analyzed the sero-profiles of all three species.

## Methods

### Bat sera collection

Blood samples were collected and processed at the collection sites from *P. rufus* (*n* = 115), *E. dupreanum* (*n* = 456), and *R. madagascariensis* (*n* = 534) bats in Madagascar from 2014 to 2020 as part of an ongoing longitudinal study following previously published methods [26–28]. Blood samples were centrifuged for 15 min at 6000 rpms in the field to separate red blood cell pellets from sera. All sampling was carried out in strict accordance with research permits obtained from the Madagascar Ministry of Forest and the Environment (permit numbers 019/18, 170/18, 007/19, 197/19, 14/20) and under guidelines posted by the American Veterinary Medical Association. All field protocols employed were pre-approved by the UC Berkeley Animal Care and Use Committee (ACUC Protocol # AUP-2017-10-10393), and every effort was made to minimize discomfort to animals.

### Antigen-based multiplex microsphere immunoassay

With the exclusion of the MARV surface glycoprotein (GP), recombinant filovirus GP were expressed as soluble, native-like trimeric ectodomains in a mammalian cell-culture system (FreeStyle 293 Expression System, Thermo Fischer Scientific; Waltham, MA) and purified as previously described [29–31]. MARV GP was expressed in stably transformed *Drosophila* S2 cells [32].

Purified GP antigens were coupled to magnetic microspheres following manufacturer guidelines (Luminex Corp, Austin, TX) using 15 µg of protein to 100 µL of microspheres. Serology was performed using a multiplex microsphere immunoassay against the GP of several filoviruses, including EBOV, BDBV, TAFV, SUDV, Bombali virus (BOMV), RESTV, RAVV, MLAV, and Lloviu virus (LLOV). We also added a mock protein control that was purified from the supernatant of 293-F cell lines transfected with an empty pcDNA3.1 expression vector to aid in establishing anti-GP antibody signal to noise levels (Appendix Table 1). A master mix of GP-coupled microspheres was used in a 96-well microtiter plate to test bat serum samples diluted 1:500 in 1X PBS without calcium or magnesium (Corning Inc., Corning, NY) for the presence of primarily immunoglobulin G (IgG) antibodies binding to antigens. After 45 min of agitation at room temperature, all wells were washed with PBS plus 0.05% Tween20 (PBST) and treated with the 1:1 combination of biotin-conjugated Protein A and biotin-conjugated Protein G (1:1000 of each in PBST) (Pierce, Thermo Fischer Scientific). Plates were incubated for another 45 min at room temperature with gentle agitation and washed. Then, a 1:1000 preparation of streptavidin-r-phycoerythrin (Invitrogen, Waltham, MA) in PBST was added to each well. Lastly, plates were agitated for 45 min and washed, then antigen-microspheres were resuspended in PBST. Antigen-antibody complexes were measured as a median fluorescence intensity (MFI) with a BioPlex 200 HTF multiplexing system (BioRad, Hercules, CA). Machine settings included a High RP1 target and a minimum count of 100 beads per unique antigen-bead region. Based on the initial results, the *R. madagascariensis* serum samples were tested in a second independent multiplex panel that only included MARV and RAVV GP.

**Table 1:** Summary of calculated Seroprevalence and corresponding confidence based on mixture model analysis for BDBV, MARV, and RAVV in *Rousettus Madagascariensis* bats, using cutoff of 85, 90, 95, and 99% confidence previously calculated specific to each species

Bat species	*N*	Virus Target	Sero_85_% (*N*)	Sero_90_% (*N*)	Sero_95_% (*N*)	Sero_99_% (*N*)
*Rousettus madagascariensis*	534	BDBV	5.06 (27)	4.49 (24)	3.75 (20)	1.50 (8)
		MARV	33.33 (178)	30.37 (162)	25.66 (137)	12.55 (67)
		RAVV	29.78 (159)	27.90 (149)	24.16 (129)	15.36 (82)

### Data analysis

We used principal component analysis (PCA) to reduce the dimensionality of data and k-medoid clustering to identify distinct antigen-antibody seroreactivity profiles and major virus targets of interest for each bat species (see Technical Appendix) [33–36]. Next, mixture model analysis was used to calculate MFI threshold cutoffs used to determine seropositivity for viruses identified by partitioning around medoids (PAM) (see Technical Appendix). Spearman’s rank correlation analyses were performed on MFI responses for MARV and RAVV GP MFI results, including for both all MFI values and only seropositive MFI values to eliminate the effects of negative data. Single antigen and double antigen positives as a percentage of the total bat species population were calculated using the species and antigen-specific MFI cutoffs. After determination of likely occurring virus targets, final seroprevalences were calculated using the antigen- and species-specific MFI cutoffs from mixture modeling (see Technical Appendix).

## Results

### Identification of filovirus serologic signatures

**Fig. 1:**
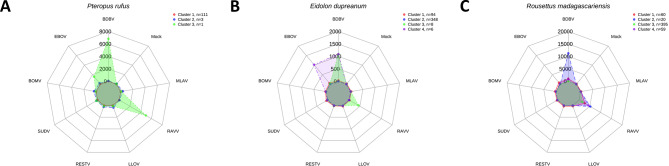
Filovirus cluster profile with corresponding average cluster antibody magnitude for all three Malagasy bat species. Radar charts comparing the serological cluster profiles of three Malagasy bat species to eight filovirus envelope glycoprotein (GP) antigens and a mock protein antigen control. The radial axes represent each of the eight filoviruses GP antigens used as the serological target for detection of antibodies. The scales are a continuous linear measurement of median fluorescence intensity (MFI). Connecting lines represent individual clusters based on k-medoids using five components. (**A**) *Pteropus rufus* (Madagascan flying foxes; *n* = 115) has three clusters, with Cluster 1 (red) and 2 (blue) representing individuals with no seroreactivity, and Cluster 3 (green) consisting of a single individual with BDBV, EBOV, and RAVV GP seroreactivity. (**B**) *Eidolon dupreanum* (Madagascan fruit bats; *n* = 456) has four clusters, with Cluster 1 (red) and 2 (blue) representing individuals with no seroreactivity, Cluster 3 (green) representing individuals with BDBV and RAVV GP seroreactivity, and Cluster 4 (purple) representing individuals with EBOV and BDBV GP seroreactivity. (**C**) *Rousettus madagascariensis* (Madagascan rousette bats; *n* = 534) has four clusters, with Cluster 1, 3, and 4 representing individuals with varying RAVV seroreactivity and Cluster 2 representing individuals with both BDBV and RAVV seroreactivity

We explored antibody profiles against filoviruses in serum samples from *P. rufus*,* E. dupreanum*, and *R. madagascariensis* bats. PCA dimensionality reduction was guided by parallel analysis, resulting in the selection of five components to account for at least 80% variance in the data (Appendix Table 2; Appendix Fig. 1). Clustering analysis highlighted distinct sero-profiles for each of the three bat species. In *P. rufus* samples, three serological clusters were identified; however, seroreactivity was observed in only one serum sample that demonstrated simultaneous seroreactivity with EBOV, BDBV, and RAVV GP (Fig. 1A). In *E. dupreanum*, a four-cluster model identified one cluster comprised of seroreactivity to both EBOV and BDBV and a second cluster comprised of seroreactivity to BDBV and RAVV (Fig. 1B). Notably, none of the observed antibody levels exceeded magnitudes of seroreactivity above 5000 MFI. Lastly, in *R. madagascariensis*, a four-cluster model resulted in one cluster (Cluster 2) with seroreactivity to both BDBV and RAVV GP, while Cluster(s) 1, 3, and 4 were specifically reactive with RAVV at varying magnitudes (Fig. 1C). EBOV, BDBV, and RAVV were identified as viruses of interest for *P. rufus* and *E. dupreanum*, and BDBV and RAVV for *R. madagascariensis* bats.

### Estimation of virus seroprevalences

We next sought to determine seropositivity cutoffs via mixture model analysis for each of the identified viruses of interest in each bat species (Appendix Fig. 2; Appendix Table 3). We observed that values for the most conservative calculated MFI cutoffs for *P. rufus* and *E. dupreanum* bats were low across all viruses of interest (EBOV, BDBV, and RAVV), with some of the cutoffs falling within corresponding MFI levels against a mock protein antigen, a proxy for immunoassay noise (Appendix Fig. 3A and B). In contrast, MFI cutoffs calculated for BDBV in the *R. madagascariensis* bats were consistently above the mock protein MFI levels (Appendix Fig. 3C). We suspect that the low cutoffs for both *P. rufus* and *E. dupreanum* reflect forced clustering of seronegative data and, consequently, represent invalid serostatus thresholds compared to seropositive cutoffs for antigen-antibody complexes detected in the *R. madagascariensis* samples. We also compared these cutoffs to our estimate for the lower limit of quantification of the Bio-Plex 200 HTF multiplexing system and selected cutoffs that were greater than 3416.21 MFI (Appendix Fig. 4). As a result, we took no further steps to identify seropositive *P. rufus* and *E. dupreanum* bats, while for *R. madagascariensis*, we calculated final BDBV seroprevalence to be 1.50–5.06% using cutoffs at 85, 90, 95, and 99% confidence level (Table 1).

**Fig. 2:**
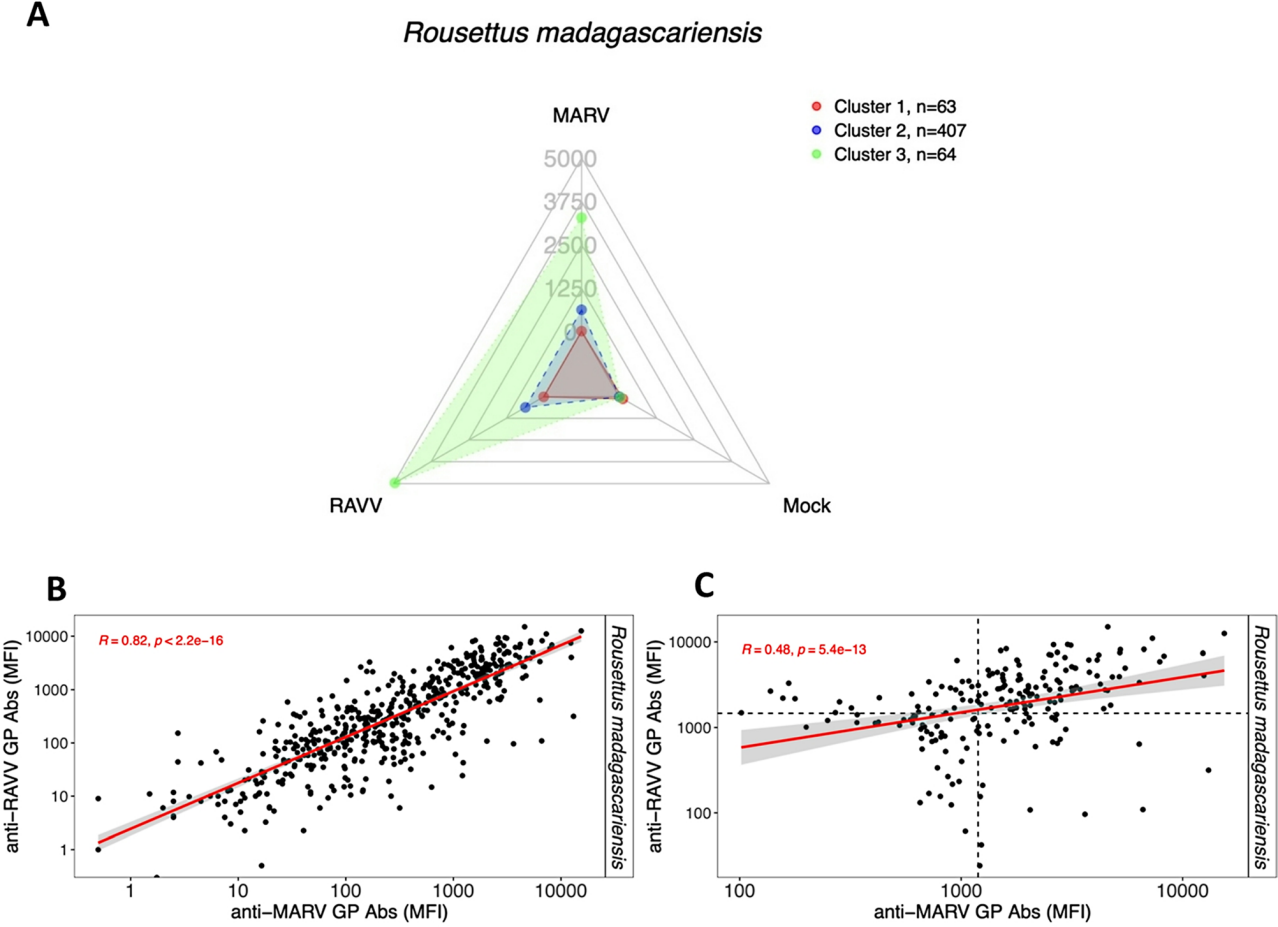
Orthomarburgvirus cluster profile with corresponding average cluster antibody magnitude for R. madagascariensis bats. Radar chart displaying clusters of serological profiles of *Rousettus madagascariensis* (Madagascan rousette bats; *n* = 534) for an orthomarburgvirus-specific panel. The radial axes represent each of the two orthomarburgvirus GP and mock antigens used as the serological target for detection of antibodies. The scales are a continuous linear measurement of median fluorescence intensity (MFI), or antibody levels. Data points and connecting lines represent individual clusters based on k-medoids. This profile contains three clusters, with Cluster 1 individuals with no seroreactivity and Cluster 2 and 3 representing individuals with varying magnitude of RAVV and MARV GP seroreactivity. Correlation comparison of antibody responses measured in MFI against RAVV and MARV for *R. madagascariensis* bats with resulting Spearman’s correlation coefficient and corresponding p-value. (**B**) Correlation comparison for all samples of *R. madagascariensis* (*n* = 534). (**C**) Correlation comparisons for seropositive samples of *R. madagascariensis* as determined by the 85% confidence (lower confidence) cutoff from mixture models analysis (*n* = 202; cutoffs MARV MFI = 642.5 & RAVV MFI = 1010.25)

Because the *R. madagascariensis* bat populations had serological reactivity against RAVV, we further investigated whether antibody profiles against MARV could be detected, as MARV GP was not included in our original antigen panel. We re-tested *R. madagascariensis* bat serum samples against an orthomarburgvirus-specific multiplex panel containing RAVV and MARV GP only. We observed different profiles of serological reactivity against both virus targets, where Cluster 2 and 3 were seroreactive with both RAVV and MARV GP and Cluster 1 was not seroreactive with either (Fig. 2A). These viruses belong to the same species (*Orthomarburgvirus marburgense*); thus, possible cross-reactions were anticipated. Correlation analyses indicated that MARV and RAVV elicited independent serological profiles, so seropositivity cutoffs were calculated for both MARV and RAVV in this population using mixture model analysis (Appendix Fig. 5; Appendix Table 4). The most conservative MFI cutoffs for MARV and RAVV GP were confirmed to be above the corresponding mock MFI values (Appendix Fig. 6). Correlation of anti-MARV GP and anti-RAVV GP antibody responses was strong (ρ = 0.82, *p* < 2.2e-16) (Fig. 2B), but correlation comparison of the seropositive groups using their respective calculated cutoffs was weak (ρ = 0.48, *p* = 5.4e-13) (Fig. 2C). There were 4.49% (24/534) RAVV – single seropositive, 8.05% (43/534) MARV – single seropositive, and 25.28% (135/534) double seropositive individual serum samples. Our results indicated that final seroprevalence estimates should be calculated for both MARV and RAVV in the sampled *R. madagascariensis* bats. Cutoffs calculated at 85%, 90%, and 95% confidence were much lower and were < 3416.21 MFI, our estimate for the lower limit of quantification (Appendix Table 4). Our seropositivity cutoffs for both MARV and RAVV that were closest to the estimate of the machine lower limit of quantification and above corresponding mock MFI values were at a 99% confidence level. Therefore, seroprevalences calculated at a conservative cutoff with a 99% confidence level were 12.55% for MARV and 15.36% for RAVV in *R. madagascariensis* bats (Table 1).

## Discussion

Our findings suggest that at least two filoviruses, one antigenically-related to both orthomarburgviruses (MARV and RAVV) and another to orthoebolaviruses (BDBV) are circulating in wild fruit bats in Madagascar. We observed BDBV and RAVV seropositivity in *R. madagascariensis* bats compared to *P. rufus* and *E. dupreanum* bats (Table 1). The relationship between *Rousettus* bat species and orthomarburgviruses has already been established with *R. aegyptiacus*, a confirmed host for MARV and RAVV [6]. In contrast, serological evidence of orthomarburgviruses in *Eidolon* bats is limited and more so for *Pteropus* bats, thus low RAVV seroprevalence observed here in these two species is not unexpected [19, 37]. Our analysis estimates that the *R. madagascariensis* bats in our study have seroprevalence ranges of 12.55–33.33% for MARV and 15.36–29.78% for RAVV (Table 1). These estimates are within the range of previously reported seroprevalences for MARV in *R. aegyptiacus*, which range from 7 to 82% [38–40]. The serological evidence of RAVV and MARV in *R. madagascariensis* bats supports predictions of *Rousettus* bats as filovirus hosts and affirms a particular association with orthomarburgviruses [20].

Additionally, BDBV seroprevalence estimates ranged from 1.50 to 5.06% in *R. madagascariensis* bats, which was lower in comparison to orthomarburgvirus seroprevalence estimates (Table 1). The lower BDBV seroprevalence can be explained by the likely possibility that the BDBV seropositive samples in our dataset reflect cross-reactivity from one or more BDBV-related viruses circulating in Malagasy fruit bat populations. These antigenically-divergent viruses would then induce antibodies that can cross-react with the BDBV GP, but at lower levels and thus, account for the lower overall detectable antibody magnitudes. Alternatively, it could be more simply that a BDBV-like virus circulates at lower prevalence in these bats. Therefore, we cannot infer conclusive BDBV – host relationships from this analysis, consistent with prior biosurveillance efforts, which have been thus far unable to identify wildlife hosts of BDBV or other orthoebolaviruses [41].

The observed EBOV and BDBV seroreactivity in *P. rufus* and *E. dupreanum* warrants additional examination, as orthoebolavirus antisera is known to be cross-reactive between closely related heterotypic viruses [42–44]. We ultimately determined to not calculate seroprevalence for orthoebolaviruses in *P. rufus* and *E. dupreanum* bats due to extremely low MFI cutoffs within the MFI noise levels of the multiplexing system, which suggested that the dataset could be entirely seronegative (Appendix Fig. 3A and B). In contrast, we retained both MARV and RAVV as viruses of interest in the *R. madagascariensis* dataset because of the low observed correlation between MARV and RAVV seropositives, and a number of single seropositives identified for either MARV or RAVV, as well as some double seropositives (Fig. 2B-C). This serological profile is likely attributable to polyclonal antisera elicited by one or more antigenically-related orthomarburgvirus(es) circulating in the natural system.

## Conclusion

Previous sero-surveillance provided the first evidence of filoviruses in bats residing in Madagascar, detecting anti-EBOV GP antibodies in *P. rufus* and *R. madagascariensis*, but not in *E. dupreanum* bats [26]. Our expanded analysis using a multiplex panel with a larger diversity of orthoebolavirus antigens suggests that the serological signatures originally detected against EBOV GP are likely more specific to a BDBV-like GP or, in the case of *R. madagascariensis*, a RAVV or MARV-like GP. The addition of orthomarburgvirus antigens to our serological panel has allowed us to detect specific seroreactivity to MARV and RAVV in *R. madagascariensis* bats for the first time. One limitation of this study is that due to the limited volume of sera we were unable to further characterize the MARV and RAVV GP seropositive samples for virus neutralizing activity. Neutralizing activity of these seropositive samples would have strengthened our assessment of the antigenic-relatedness of the orthomarburgviruses detected in the multiplex immunoassay to the prototypic viruses, MARV and RAVV.

Native Malagasy bat species are hunted and consumed on the island for bushmeat [45], a possible animal-human interface for spillovers to occur. A historical sero-survey was conducted in five areas of Madagascar investigating EBOV, SUDV, MARV, and other non-filovirus hemorrhagic fever viruses in humans. This study found 13.3% EBOV seroprevalence in humans living near Antananarivo, Madagascar’s capital city, and no evidence of MARV or SUDV [46]. Next steps for effective pandemic readiness include (i) optimizing sampling efforts to increase the likelihood of collecting samples for sequencing viral genomes to confirm the BDBV-like orthoebolavirus and to distinguish the multiple orthomarburgviruses circulating in the bat population [47–49], (ii) evaluating zoonotic potential of any resulting viral genomes, and (iii) identifying the interfaces for spillover into susceptible human populations [50].

## Supplementary Information

Below is the link to the electronic supplementary material.

Supplementary Material 1

## Data Availability

Data and key materials utilized in this study are available as a sharable resources upon reasonable request to the corresponding authors.

## Abbreviations

EBOV: Ebola virus
BDBV: Bundibugyo virus
SUDV: Sudan virus
TAFV: TaÏ forest virus
BOMV: Bombali virus
RESTV: Reston virus
MARV: Marburg virus
RAVV: Ravn virus
MLAV: Měnglà virus
DEHV: Dehong virus
LLOV: Lloviu virus
PCA: Principal component analysis
MFI: Median fluorescence intensity
