# Supplementary material for: Serologic evidence of orthomarburgviruses and an orthoebolavirus in frugivorous Malagasy bats
Source: One Health Outlook. 2025 Oct 30;7:55. doi: 10.1186/s42522-025-00178-0 (PMC12577238; doi:10.1186/s42522-025-00178-0)
Supplement: Supplementary file 1 — Supplementary Material 1 [file 42522_2025_178_MOESM1_ESM.docx]

**TECHNICAL APPENDIX**

**Supplementary Methods**

***Clustering Approaches to Identify Filovirus Sero-profiles***

The R function ‘prcomp’ was used to perform a principal components analysis (PCA) on a data matrix representing the MFI values for the nine protein antigens in our filovirus panel, data from the MARV/RAVV GP retest were analyzed independently by PCA. The cumulative proportion of variance for each component was evaluated and it was observed that principal components 1 through 5 accounted for more than 80% of the variation in the data (Appendix Table 2). PCA was used to account for data sparsity in immunofluorescence values with the retention of five dimensions as determined by parallel analysis [1] (Appendix Figure 1). The silhouette method and elbow method were used to select the k-medoids number for clustering using the package “factoextra,” with a suggested 2 – 6 clusters. To further profile the serology data, the *stats* package in R software [2] and *factoextra* [3] were used. Negative MFI results were zeroed and the data were scaled and centered prior to dimensionality reduction. Preliminary data analysis indicated that log transformation did not significantly improve the distribution in most of the data; thus, to maintain the most direct link between cluster assignment and source data, we retained data untransformed for PCA and cluster analysis.

Partitioning around medoids, or K-medoids clustering, was used to determine virus antigen-antibody profiles across all eight virus protein antigen targets, mock protein excluded [4, 5]. Optimized average silhouette width predefined the number of clusters which were confirmed as resolved using gap statistic values with respect to correlation to biological interpretation of phenotypical group [6]. Optimal cluster counts were bounded between 3 and 6 clusters to select for greater nuance than binary classification and limit redundant cluster profiles.

***Mixture Model Analysis***

For the calculation of seroprevalence for all identified targets, we used the R package *mclust* for univariate, Gaussian-based mixture modelling to identify seropositivity cutoffs that were species and antigen-specific. Prior to analysis, MFI values <1 were converted to 1, then log10 transformed for input into mixture model analysis. We allowed *mclust* to select the optimal cluster number between 1 – 9 clusters to effectively represent the distribution of MFI data for each antigen and bat species combination, under assumptions of “E” (equal) variance. Cutoffs were calculated at 85, 90, 95, and 99% confidence levels. Our analysis further assumed that that i) the MFI results from a single antigen for a single species spanned a range of values that could be represented by a finite number (1-9) of Gaussian (normal) distributions, ii) the best-fit model identified by the analysis resulted in subpopulations (clusters) reflective of the whole distribution of antigen-antibody levels (MFI), and iii) the highest-value cluster represented seropositive individuals. The assumption that the highest value cluster represents seropositive individuals is a conservative assumption. In serological datasets testing well-established host-antigen relationships, two to three gaussian clusters are typically observed, with a lower distribution corresponding to seronegative individuals and a higher distribution corresponding to seropositive individuals [7, 8]. In the absence of standardized controls to establish immunoassay thresholds for as-of-yet undescribed viruses, we elected to instead model the entire range of MFI clusters in the dataset. When many serological clusters are observed in a dataset, these are typically interpreted to represent a range of exposure histories [9, 10]: while lowest value clusters likely correspond to seronegative status, mid-range value clusters may indicate waning antibodies to less recent antigen exposures or cross-reactivity in antibodies raised against unknown viruses with known antigens included in our dataset. Nonetheless, the highest cluster is the most likely to represent more recent infection-induced antibodies, making this the most reliable metric for calculation of seroprevalence. Indeed, we observed a wide range of optimal cluster numbers among the three fruit bat species and their respective antigens, with fewer clusters observed for host-antigen relationships for which we felt the most confident (e.g. *R. madagascariensis* BDBV and RAVV; Appendix Figure 2).

To account for the limitation that modeled cutoffs can still identify highest value distributions within largely negative immunoassay data, mixture model threshold cutoffs at all four confidence levels were examined in relationship to both mock protein antigen representation of noise levels and against the linear range of a four – parametric logistic (4-PL) regression curve fit model for the detection of anti-MARV antibodies, primarily IgG, in this immunoassay utilizing a Bio-Plex 200 HTF multiplexing system (Appendix Figure 6). The 4-PL curve fitting model is a standard approach to identify the linear slope of immunoassays and has been used by our group for both SARS-CoV-2 and Ebola virus multiplex immunoassays [11-13]. Therefore, through this combined mixture model- and immunoassay-driven strategy, we decided on the most appropriate confidence cutoffs specific to each species and antigen, which for BDBV in *R. madagascariensis* included the full range of 85, 90, 95, and 99% confidence cutoffs, but for MARV and RAVV in *R. madagascariensis* was only the 99% confidence cutoff. We conclude that lower cutoffs within the mock noise levels and/or outside of the lower quantification limit (MFI < 3416.21) of the linear slope of anti-MARV GP 4-PL curve are likely reflective of clustering of negative data for the first two species, *P. rufus* and *E. dupreanum*.

**REFERENCES**

1. Franklin SB, Gibson DJ, Robertson PA, Pohlmann JT, Fralish JS. Parallel Analysis: a method for determining significant principal components. Journal of Vegetation Science. 1995;6(1):99-106; doi: <https://doi.org/10.2307/3236261>.

2. R Core Team: R: A language and environment for statistical computing. In. Vienna, Austria: R Foundation for Statistical Computing; 2021.

3. Kassambara A, Mundt F: Factoextra: Extract and Visualize the Results of Multivariate Data Analyses. . In., 1.0.7 edn; 2020.

4. Hartigan JA, Wong MA. A K-Means Clustering Algorithm. Journal of the Royal Statistical Society Series C: Applied Statistics. 2018;28(1):100-8; doi: 10.2307/2346830.

5. Partitioning Around Medoids (Program PAM). In: Finding Groups in Data. 1990. p. 68-125.

6. Dalmaijer ES, Nord CL, Astle DE. Statistical power for cluster analysis. BMC Bioinformatics. 2022;23(1):205; doi: 10.1186/s12859-022-04675-1.

7. Yang DA, Laven RA. Performance of the StaphGold ELISA test in determining subclinical Staphylococcus aureus infections in dairy cows using a Gaussian mixture model. Veterinary Medicine and Science. 2022;8(4):1632-9; doi: <https://doi.org/10.1002/vms3.785>.

8. Tessier E, Litt D, Ribeiro S, Abdul Aziz N, Campbell H, Amirthalingam G, et al. Mixture modelling of Bordetella pertussis serology samples to evaluate anti-pertussis toxin immunoglobulin G titre thresholds for positivity: England 2008–2022. Journal of Medical Microbiology. 2024;72(12); doi: <https://doi.org/10.1099/jmm.0.001774>.

9. Rota MC, Massari M, Gabutti G, Guido M, De Donno A, Atti MLCd. Measles serological survey in the Italian population: Interpretation of results using mixture model. Vaccine. 2008;26(34):4403-9; doi: <https://doi.org/10.1016/j.vaccine.2008.05.094>.

10. Fornace KM, Senyonjo L, Martin DL, Gwyn S, Schmidt E, Agyemang D, et al. Characterising spatial patterns of neglected tropical disease transmission using integrated sero-surveillance in Northern Ghana. PLOS Neglected Tropical Diseases. 2022;16(3):e0010227; doi: 10.1371/journal.pntd.0010227.

11. Goguet E, Olsen CH, Meyer WA, 3rd, Ansari S, Powers JH, 3rd, Conner TL, et al. Immune and behavioral correlates of protection against symptomatic post-vaccination SARS-CoV-2 infection. Front Immunol. 2024;15:1287504; doi: 10.3389/fimmu.2024.1287504.

12. Laing ED, Weiss CD, Samuels EC, Coggins SA, Wang W, Wang R, et al. Durability of Antibody Response and Frequency of SARS-CoV-2 Infection 6 Months after COVID-19 Vaccination in Healthcare Workers. Emerg Infect Dis. 2022;28(4):828-32; doi: 10.3201/eid2804.212037.

13. Roe MD, Hood G, Sterling SL, Yan L, Boré JA, Tipton T, et al. Performance of an envelope glycoprotein-based multiplex immunoassay for Ebola virus antibody detection in a cohort of Ebola virus disease survivors. J Virol Methods. 2025;331:115057; doi: 10.1016/j.jviromet.2024.115057.

**Appendix Table 1. Summary of Mock MFI responses specific to each species that represent antibody-antigen noise levels.**

|  | *P. rufus* | *E. dupreanum* | *R. madagascariensis* | *R. madagascariensis (orthomarburgvirus -panel only)* |
| --- | --- | --- | --- | --- |
| Total n | 115 | 456 | 534 | 534 |
| Mean | 16.95 | 12.93 | 120.85 | 37.38 |
| Median | 7.00 | 7.50 | 29.63 | 20.88 |
| Standard deviation | 45.25 | 25.81 | 331.81 | 60.78 |
| Max | 440.00 | 276 | 2836.50 | 646.75 |
| Min | -12.00 | -37.00 | -6.50 | -38.00 |

**Appendix Table 2. Summary of eigenvalues, variance (%), and cumulative variance (%) for principal component analysis of each Malagasy bat species antibody results.**

|  | *Pteropus rufus* | | | *Eidolon dupreanum* | | | *Rousettus madagascariensis* | | |
| --- | --- | --- | --- | --- | --- | --- | --- | --- | --- |
|  | Eigenvalue | Variance (%) | Cumulative Variance (%) | Eigenvalue | Variance (%) | Cumulative Variance (%) | Eigenvalue | Variance (%) | Cumulative Variance (%) |
| Dim.1 | 4.86 | 60.47 | 60.47 | 2.31 | 31.17 | 31.17 | 3.39 | 42.47 | 42.47 |
| Dim.2 | 1.63 | 20.24 | 80.71 | 1.36 | 18.37 | 49.55 | 1.49 | 18.73 | 61.20 |
| Dim.3 | 0.81 | 10.09 | 90.80 | 0.95 | 12.82 | 62.37 | 1.07 | 13.45 | 74.65 |
| Dim.4 | 0.43 | 5.41 | 96.22 | 0.85 | 11.50 | 73.86 | 0.59 | 7.42 | 82.07 |
| Dim.5 | 0.17 | 2.06 | 98.28 | 0.71 | 9.53 | 83.40 | 0.55 | 6.84 | 88.91 |
| Dim.6 | 0.09 | 1.07 | 99.35 | 0.47 | 6.40 | 89.79 | 0.40 | 5.03 | 93.93 |
| Dim.7 | 0.02 | 0.29 | 99.64 | 0.29 | 3.90 | 93.69 | 0.25 | 3.14 | 97.07 |
| Dim.8 | 0.02 | 0.22 | 99.85 | 0.25 | 3.41 | 97.11 | 0.20 | 2.50 | 99.57 |
| Dim.9 | 0.01 | 0.15 | 100.00 | 0.21 | 2.89 | 100.00 | 0.03 | 0.43 | 100.00 |


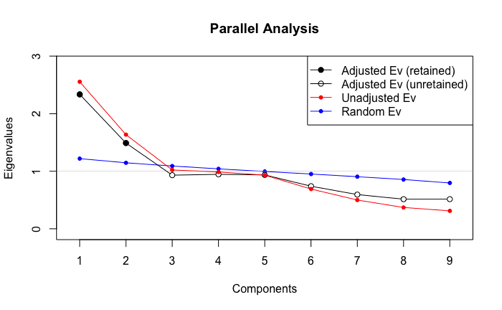

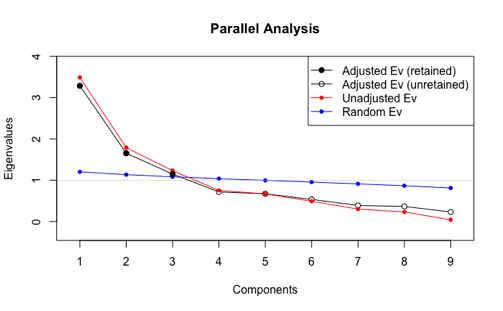

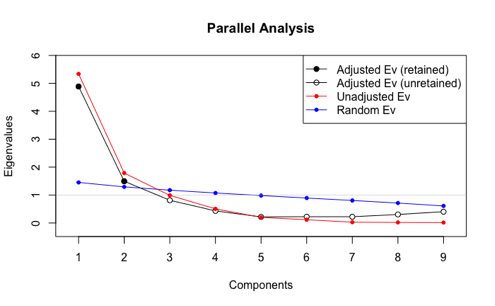


**Appendix Figure 1. Parallel Analysis of Skree plots**

Parallel analysis graphs of eigenvalues associated with each principal components for (Left) *Pteropus rufus*, (Middle) *Eidolon dupreanum*, and (Right) *Rousettus madagascariensis*, comparing calculated eigenvalues to simulated eigenvalues.


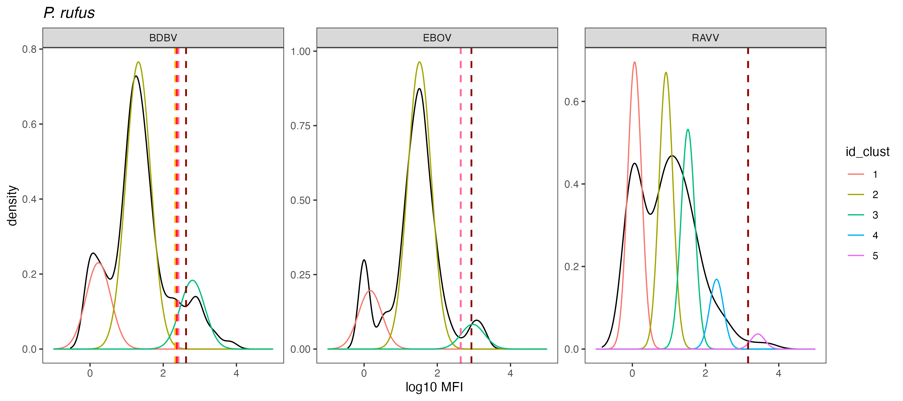

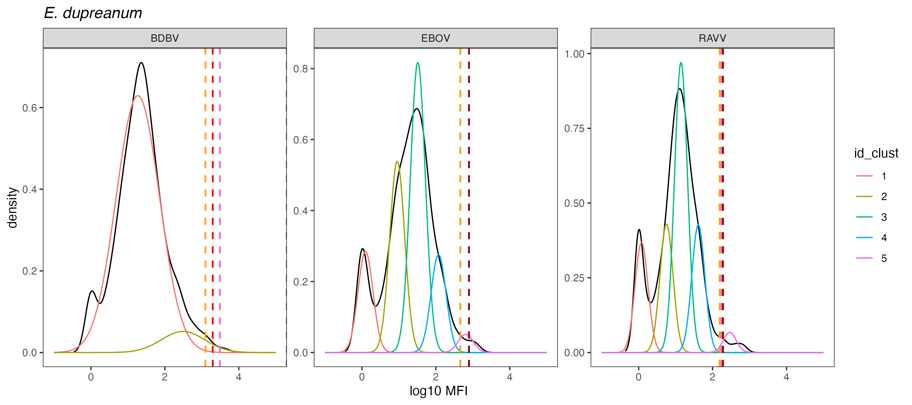

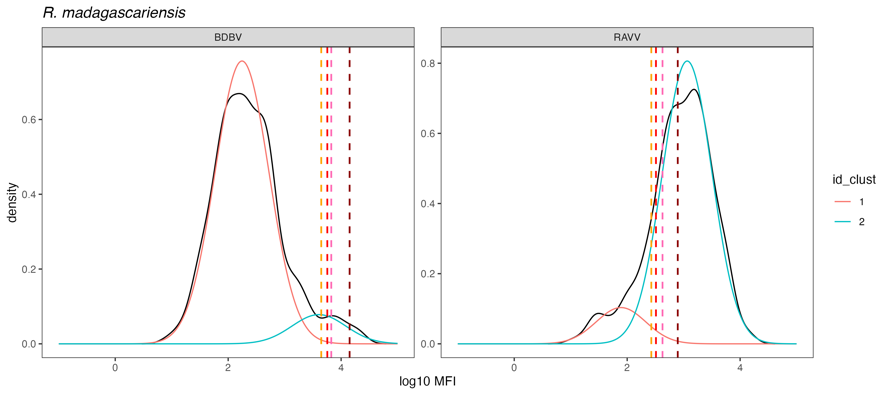
**Appendix Figure 2. Mixture model density plots with MFI cutoffs for all three Malagasy bat species.** Mixture model density plots showing cluster distribution across transformed MFI results for each specific antigen and bat species combination and resulting MFI cutoffs at 85% (orange dotted line), 90% (red dotted line), 95% (hot pink dotted line), and 99% (dark red dotted line) confidence. (A) *P. rufus* mixture model density plot identified best fit with multiple clusters within the distribution of MFI results for BDBV (3), EBOV (3), and RAVV (5) GP. (B) *E. dupreanum* mixture model density plot identified best fit with multiple clusters within the distribution of MFI results for BDBV (2), EBOV (5), and RAVV (5) GP. (C) *R. madagascariensis* mixture model density plot identified best fit with 2 clusters within the distribution of MFI results for BDBV and RAVV GP.

**B**

**C**

**A**

**Appendix Table 3. Mixture model clusters and seropositivity cutoffs (85, 90, 95, and 99% confidence interval) for EBOV, BDBV, and RAVV for *P. rufus* & *E. dupreanum*, and for BDBV and RAVV for *R. madagascariensis*.**

| **Bat species** | **Antigen** | **Cluster N** | **Cluster ID** | **prop** | **Mean** | **Standard Deviation** | **85% Confidence Cutoff**  **(MFI)** | **90% Confidence Cutoff**  **(MFI)** | **95% Confidence Cutoff (MFI)** | **99% Confidence Cutoff (MFI)** |
| --- | --- | --- | --- | --- | --- | --- | --- | --- | --- | --- |
| *Pteropus rufus* | EBOV | 3 | 1 | 0.16 | 0.17 | 0.32 | 438.00 | 438.00 | 438.00 | 856.25 |
|  |  |  | 2 | 0.78 | 1.51 | 0.32 |  |  |  |  |
|  |  |  | 3 | 0.07 | 2.97 | 0.32 |  |  |  |  |
|  | BDBV | 3 | 1 | 0.20 | 0.23 | 0.34 | 213.50 | 234.25 | 261.75 | 419.75 |
|  |  |  | 2 | 0.65 | 1.32 | 0.34 |  |  |  |  |
|  |  |  | 3 | 0.16 | 2.80 | 0.34 |  |  |  |  |
|  | RAVV | 5 | 1 | 0.33 | 0.06 | 0.19 | 1452.75 | 1452.75 | 1452.75 | 1452.75 |
|  |  |  | 2 | 0.32 | 0.92 | 0.19 |  |  |  |  |
|  |  |  | 3 | 0.25 | 1.52 | 0.19 |  |  |  |  |
|  |  |  | 4 | 0.08 | 2.30 | 0.19 |  |  |  |  |
|  |  |  | 5 | 0.02 | 3.43 | 0.19 |  |  |  |  |
| *Eidolon dupreanum* | EBOV | 5 | 1 | 0.14 | 0.10 | 0.20 | 459.50 | 459.50 | 788.50 | 788.50 |
|  |  |  | 2 | 0.27 | 0.96 | 0.20 |  |  |  |  |
|  |  |  | 3 | 0.42 | 1.51 | 0.20 |  |  |  |  |
|  |  |  | 4 | 0.14 | 2.07 | 0.20 |  |  |  |  |
|  |  |  | 5 | 0.03 | 2.81 | 0.20 |  |  |  |  |
|  | BDBV | 2 | 1 | 0.92 | 1.27 | 0.59 | 1250.00 | 1972.50 | 3079.00 | Inf |
|  |  |  | 2 | 0.08 | 2.49 | 0.59 |  |  |  |  |
|  | RAVV | 5 | 1 | 0.16 | 0.08 | 0.18 | 154.50 | 154.50 | 174.00 | 191.00 |
|  |  |  | 2 | 0.19 | 0.75 | 0.18 |  |  |  |  |
|  |  |  | 3 | 0.43 | 1.15 | 0.18 |  |  |  |  |
|  |  |  | 4 | 0.19 | 1.61 | 0.18 |  |  |  |  |
|  |  |  | 5 | 0.03 | 2.46 | 0.18 |  |  |  |  |
| *Rousettus madagascariensis* | BDBV | 2 | 1 | 0.91 | 2.25 | 0.48 | 4446.00 | 5694.00 | 6691.50 | 14140.50 |
|  |  |  | 2 | 0.09 | 3.61 | 0.48 |  |  |  |  |
|  | RAVV | 2 | 1 | 0.11 | 1.89 | 0.44 | 267.50 | 323.50 | 422.50 | 786.50 |
|  |  |  | 2 | 0.89 | 3.06 | 0.44 |  |  |  |  |


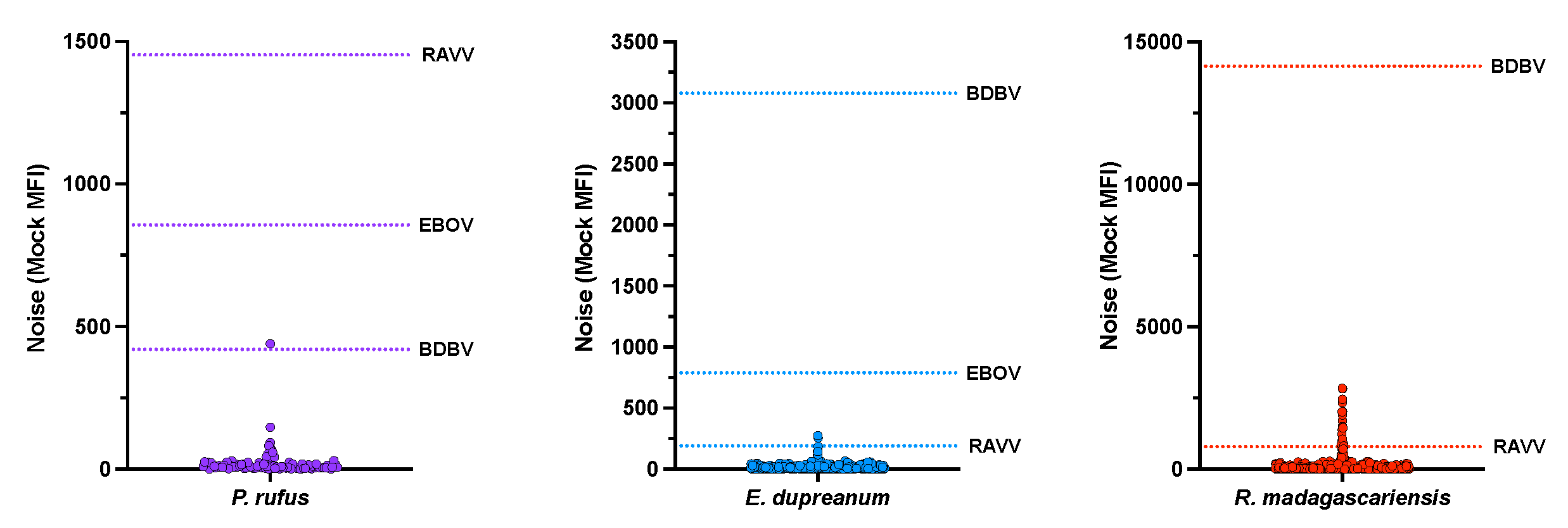


**C**

**B**

**A**

**Appendix Figure 3. Visualization of species – specific calculated cutoffs to corresponding species mock protein antigen MFI values representing anti – GP antibody noise levels.**

Mock protein antigen MFI values for each bat species with corresponding most conservative MFI cutoffs for identified virus antigens. (A) *P. rufus* plotted Mock MFI values with MFI cutoffs for EBOV GP = 856.25, BDBV GP = 419.75, and RAVV GP = 1452.75. (B) *E. dupreanum* plotted Mock MFI values with MFI cutoffs for EBOV GP = 788.50, BDBV GP = 3079.00, and RAVV GP = 191.00. (C) *R. madagascariensis* plotted Mock MFI values with MFI cutoffs for BDBV GP = 14140.50 and RAVV GP = 786.50.

**Appendix Figure 4. A four parametric regression curve fit model was used to establish the linear range of detectable anti-Marburg virus antibodies and lower limits of quantification.** A serum sample from a non-human primate experimentally challenged with Marburg virus (MARV) was tested at eight dilutions, 2-fold, ranging from 0.038 – 5.000 ug/mL. A solid black line represents the curve fit model and the dashed lines represent the 95% confidence error from seven independent replicates. The estimated lower limit of quantification, < 3416.21 MFI, median fluorescence intensity, is indicated.


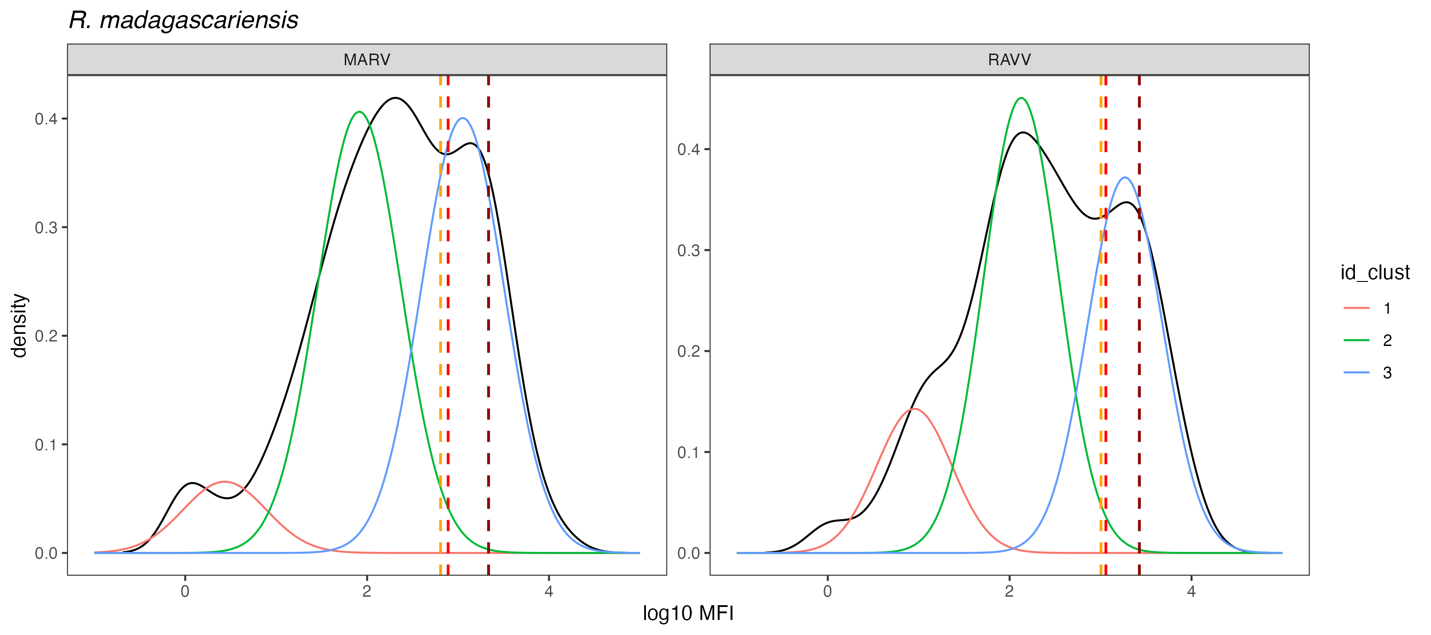


**Appendix Figure 5. Mixture models density plots calculating MFI cutoffs for MARV and RAVV for *R. madagascariensis* bats.**

Mixture model density plots showing cluster distribution across transformed MFI results for MARV and RAVV GP and resulting MFI cutoffs at 85% (orange dotted line), 90% (red dotted line), and 99% (dark red dotted line) confidence. Best-fit model identified 3 clusters within the distribution of MFI results for MARV and RAVV GP.

**Appendix Table 4. Mixture model clusters and positivity cutoffs (85, 90, 95, and 99% confidence) for MARV and RAVV for *R. madagascariensis*.**

| **Bat species** | **Antigen** | **Cluster N** | **Cluster ID** | **prop** | **Mean** | **Standard Deviation** | **85% Confidence Cutoff**  **(MFI)** | **90% Confidence Cutoff**  **(MFI)** | **95%**  **Confidence Cutoff (MFI)** | **99% Confidence Cutoff (MFI)** |
| --- | --- | --- | --- | --- | --- | --- | --- | --- | --- | --- |
| *Rousettus madagascariensis* | MARV | 3 | 1 | 0.08 | 0.43 | 0.46 | 642.50 | 777.75 | 1082.75 | 2164.00 |
|  |  |  | 2 | 0.47 | 1.91 | 0.46 |  |  |  |  |
|  |  |  | 3 | 0.46 | 3.05 | 0.46 |  |  |  |  |
|  | RAVV | 3 | 1 | 0.15 | 0.95 | 0.41 | 1010.25 | 1142.50 | 1489.00 | 2669.25 |
|  |  |  | 2 | 0.47 | 2.13 | 0.41 |  |  |  |  |
|  |  |  | 3 | 0.39 | 3.27 | 0.41 |  |  |  |  |

**Appendix Figure 6. Visualization of *Rousettus madagascariensis* calculated MFI cutoffs for RAVV and MARV GP to corresponding species Mock MFI values representing anti – GP antibody noise levels.**

Plotted Mock MFI values that resulted from the orthomarburgvirus-specific panel retest (RAVV GP, MARV GP, and Mock control protein) of *R. madagascariensis* bat samples, including the corresponding 99% confidence MFI cutoff, with RAVV GP = 2669.25 and MARV GP = 2164.00.
